# Methicillin-resistant *Staphylococcus aureus *and *Acinetobacter baumannii *on computer interface surfaces of hospital wards and association with clinical isolates

**DOI:** 10.1186/1471-2334-9-164

**Published:** 2009-10-01

**Authors:** Po-Liang Lu, LK Siu, Tun-Chieh Chen, Ling Ma, Wen-Gin Chiang, Yen-Hsu Chen, Sheng-Fung Lin, Tyen-Po Chen

**Affiliations:** 1 Department of Internal Medicine, Kaohsiung Medical University Hospital, Taiwan, Republic of China; 2 Department of Laboratory Medicine, Kaohsiung Medical University Hospital, Taiwan, Republic of China; 3 Graduate Institute of Medicine, College of Medicine, Kaohsiung Medical University, Taiwan, Republic of China; 4 Division of Clinical Research, National Health Research Institute, Taiwan, Republic of China

## Abstract

**Background:**

Computer keyboards and mice are potential reservoirs of nosocomial pathogens, but routine disinfection for non-water-proof computer devices is a problem. With better hand hygiene compliance of health-care workers (HCWs), the impact of these potential sources of contamination on clinical infection needs to be clarified.

**Methods:**

This study was conducted in a 1600-bed medical center of southern Taiwan with 47 wards and 282 computers. With education and monitoring program of hand hygiene for HCWs, the average compliance rate was 74% before our surveillance. We investigated the association of methicillin-resistant *Staphylococcus aureus *(MRSA), *Pseudomonas aeruginosa *and *Acinetobacter baumannii*, three leading hospital-acquired pathogens, from ward computer keyboards, mice and from clinical isolates in non-outbreak period by pulsed field gel electrophoresis and antibiogram.

**Results:**

Our results revealed a 17.4% (49/282) contamination rate of these computer devices by *S. aureus*, *Acinetobacter *spp. or *Pseudomonas *spp. The contamination rates of MRSA and *A. baumannii *in the ward computers were 1.1% and 4.3%, respectively. No *P. aeruginosa *was isolated. All isolates from computers and clinical specimens at the same ward showed different pulsotypes. However, *A. baumannii *isolates on two ward computers had the same pulsotype.

**Conclusion:**

With good hand hygiene compliance, we found relatively low contamination rates of MRSA, *P. aeruginosa *and *A. baumannii *on ward computer interface, and without further contribution to nosocomial infection. Our results suggested no necessity of routine culture surveillance in non-outbreak situation.

## Background

In developed countries, computers are used in the bedside area for multiple functions, including ordering, checking laboratory and image results, recording patients' conditions, and accounting. Moreover, most computer devices, such as keyboards and mice, in many countries are not water-proof and not specially designed for hospital disinfection needs. Therefore, there is a good possibility that computer interface surfaces may serve as reservoirs for nosocomial pathogens. Besides, the rate of hand washing compliance in healthcare institutions is low (~40%), which is presumably related to the contamination of inanimate surfaces of medical equipments and hospital environment with nosocomial pathogens [1]. Studies have shown that the hands or gloves of healthcare workers (HCWs) can be contaminated after touching inanimate objects in patient rooms or after touching environmental surfaces near patients [2-4].

One study reported that microbial contamination of computer interface surfaces was so prevalent that various microorganisms were isolated from more than 50% of the keyboards of hospital computers [5]. The levels of contamination varied with the proximity to the patients, the texture of inanimate surfaces and the frequency of contact. The hospital ward computer is found being less likely to be contaminated than bedside computers [6]. Schultz et al. have reported that 95% of keyboards in close proximity to patient sites had bacterial contamination. However, only 5% of these were pathogens known to be associated with nosocomial transmission [7]. Most previous studies have reported the contamination of computer interface surfaces by potential pathogens such as Methicillin-resistant *Staphylococcus aureus *(MRSA) [3,8]and *Acinetobacter baumannii *[9], but few have studied the relationship between contamination of the ward computers and clinical isolates in hospitals with improved hand hygiene compliance and during a non-outbreak period. Clinically, *A. baumannii*, *P. aeruginosa*, and MRSA cause the most common nosocomial infections and their presence correlates with environmental surface contamination [10-12]. We conducted a hospital-based surveillance study of these three important pathogens on computer interface surfaces in different ward settings and then examined the relationship of contaminated computer interface surfaces with the presence of clinical isolates in these wards during a non-outbreak period.

## Methods

We conducted a cross-sectional surveillance for *S. aureus*, *Pseudomonas *species and *Acinetobacter *species on the keyboards and mice of computers in all ward stations of Kaohsiung Medical University Hospital, a 1600-bed tertiary referral hospital that contained various speciality departments, in July 2006. The three organisms are among the most common causes of nosocomial infection in the study site where Vancomycin resistant enterococcus (VRE) accounted for less than 1% enterococcus clinical isolates. Clinical isolates of MRSA, *P. aeruginosa*, and *A. baumannii *were recovered two weeks before or after the day of the cross-sectional surveillance, when computer-associated bacteria were collected. We selected clinical and computer isolates of the same species in the same ward for comparison by antibiograms and pulsed field gel electrophoresis (PFGE) typing.

All medical records and ordering systems were computerized in this hospital. No routine disinfection protocol had been established for computer equipment. The keyboards were not covered. No routine cleaning for the surfaces of computer interfaces was performed. Hand hygiene compliance has been continuously educated and monitored every three months with method as previously described [13] by members in infection control room and the department of nursing. Every week, HCWs' hand hygiene compliance was monitored for 30 minutes in each ward. The mean of the rates from four times of monitoring was regarded as the hand hygiene compliance rate of every ward.

A sterile swab (CultureSwab Transport System, Difco, Detroit, MI) moistened with sterile saline solution was moved over the keys of keyboards and the buttons of computer mice. Then the swabs were added to brain heart infusion broth medium for 48 h at 37°C. The inoculated broth was subcultured onto blood agar plates (BBL, Cockeysville, MD, USA) and Mac-Conkey agar plates (BBL, Cockeysville, MD, USA). Organisms were identified using standard methods and the API Identification System (bioMe'rieux, Marcy l'Etoile, France). Isolates of *Pseudomonas *and *Acinetobacter *were identified to species level. We used the coagulase test (Coagulase Plasma System; Difco) to identify *S. aureus *strains. MRSA was preliminarily detected by its characteristic growth on mannitol salt agar supplemented with oxacillin (4 μg/mL). All suspected MRSA isolates were inoculated onto Mueller-Hinton agar (Becton Dickinson Microbiology Systems) supplemented with 6 μg/ml oxacillin and 4% NaCl. Isolates identified as *P. aeruginosa*, *A. baumannii*, and MRSA were further tested for antimicrobial susceptibility and for PFGE typing.

Antimicrobial susceptibility was determined by the agar diffusion method, according to the CLSI guideline [14]. Pulsed field gel electrophoresis (PFGE) was performed as described previously [15,16]. Restriction enzymes were used for identification, *Sma*I for MRSA and *Apa*I for *Acinetobacter *spp. The band patterns were visually compared and classified as indistinguishable (no difference), closely related (clonal variants, one to three band differences), possibly related (four to six band differences), and unrelated (more than six band differences), according to the criteria previously described [17]. To identify PFGE polymorphisms, each sample was analyzed by Molecular Analyst Fingerprinting, Fingerprinting Plus, and Fingerprinting DST Software (Bio-Rad Laboratories, Richmond, CA, USA). After calculation of similarities for every pair of organisms using Pearson correlation coefficients, we used the grouping method to deduce a dendrogram from the matrix via the Unweighted Pair Group Method using Arithmetic Average (UPGMA) clustering technique.

Statistics were run with SPSS software version 13.0 (SPSS Inc., Chicago, IL, USA). *P *values were calculated by the Chi-Square test for categorical variables. All tests were two-tailed, and *p *< 0.05 was considered significant.

## Results

We screened 282 computer samples that each had a keyboards and a mouse device in 47 ward stations for *S. aureus*, *Pseudomonas *spp. and *Acinetobacter *spp. Twelve of the ward stations were from intensive care units (ICUs) and 35 were from Non-ICU wards One hundred and forty-four samples were from ICU computers and 420 from ward computers. All nurse stations had six desktop computers, one for accounting, one for radiological images, and four for ordering and laboratory data checking. Although the computers for accounting were mainly operated by accountants without direct contact with patients, the other HCWs accessed these computers occasionally when the accountants were off duty. The average compliance rate was 74% in the month that the cross-sectional surveillance on computer underwent. There was no significant difference on compliance rates among wards.

Before final species identification, we found 18 isolates of *S. aureus*, 17 isolates of *Pseudomonas *spp., and 22 isolates of *Acinetobacter *spp. from 49 computer interface samples. Three computers had positive isolation from both keyboard and mouse device. Five computer interfaces had two different species identified. The overall computer contamination rate of the above three organisms is 17.4% (49/282).

For *Acinetobacter *isolates, 12 were *A. baumannii*, 7 were *A. lwoffii*, and 3 were *A. junii*. For *S. aureus *isolates, 3 were MRSA and 15 were MSSA. For *Pseudomonas *isolates, there were 12 *P. putida *isolates, 1 *P. alcaligenes*, 4 *P. stutzeri*, but none was *P. aeruginosa*. The computer contamination rate for *A. baumannii *was 4.3% (12/282) and the rate for MRSA was 1.1% (3/282) (Table 1). The combined contamination rate of either MRSA or *A. baumannii *is 5.3% (15/282).

**Table 1:** Culture results from computer interface surfaces.

	**Keyboard (+) only**	**Mouse (+) only**	**Both keyboard and mouse (+)**	**Both keyboard and mouse (-)**
	
Any of *Staphylococcus *spp., *Pseudomonas *Spp. and *Acinetobacter *spp.	30	16	3	233
	
				
	
MRSA	2	1	0	279
*A. baumannii*	8	4	0	270
Any of MRSA and *A. baumannii*	10	5	0	267

There was no significant difference about contamination rate by any of *S. aureus*, *Pseudomonas *spp. and *Acinetobacter *spp. between non-ICU (16.7%, 35/210) and ICU (19.4%, 14/72) computers (*p *= 0.591) and between accounting, radiology and order computers (*p *= 0.699). When the radiology and order computers were grouped together into the "clinical use" category, there was no significant difference in the contamination rate between accounting (10/47) and "clinical use" (39/235) computers (*p *= 0. 439). No significant higher rate of contamination of any of the above three between keyboard and mouse (*p *= 0.474) but there is a trend that the occurrence of keyboard contamination is associated with the occurrence of mouse device contamination (*p *= 0.054) by McNemar test.

When only considering MRSA, *P. aeruginosa*, and *A. baumannii*, all the differences between non-ICU and ICU computers (*p *= 0.476), keyboard and mouse specimens (*p *= 0.191), and accounting and "clinical use" computers (*p *= 0.722), were not significant (Table 2).

**Table 2:** Culture results of MRSA and *A. baumannii *isolated from computer interface surfaces according to type of computer interface, type of ward, and computer function.

(+) for MRSA or *A. baumannii*	Keyboard (+) only	Mouse (+) only	Both keyboard and mouse (+)	Both keyboard and mouse (-)
	
Ward function				
	
Non-ICU (N = 210)	6	4	0	200
ICU (N = 72)	4	1	0	67
				
	
Computer function				
	
Accounting use (N = 47)	1	2	0	44
Clinical use (N = 235)	9	3	0	223

ICU: intensive care unit

**Figure 1:**
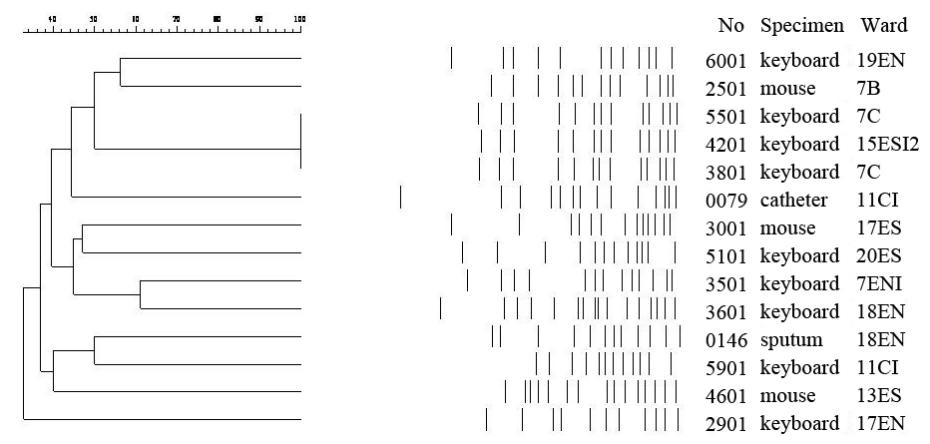
**Similarity of PFGE patterns of *A. baumannii *isolated in hospital wards, calculated by the unweighted pair group method using arithmetic averages (UPGMA)**. Similarities >70% represent the clonal spread of strains. Isolate number, source of specimens and the ward number are listed in the right side of the figure.

**Figure 2:**
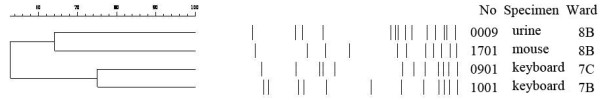
**Similarity of PFGE patterns of MRSA isolated in hospital wards, calculated by UPGMA**. Similarities >70% represent the clonal spread of strains. Isolate number, source of specimens and the ward number are listed in the right side of the figure.

Twenty isolates of *A. baumannii*, 34 isolates of MRSA, and 97 isolates of *P. aeruginosa *were identified from clinical specimens in the same hospital two weeks before and after the computer surveillance day. One MRSA isolate and two *A. baumannii *were isolated from patients of the wards where computer devices were also contaminated with MRSA and *A. baumannii*. We compared the clinical and computer isolates by antibiograms and PFGE typing. The antibiogram results of the *A. baumannii *isolated from clinical and computer specimens in ward 18EN were different on fewer than three classes of antimicrobial agents. For the other groups of MRSA and *A. baumannii *isolates, the antibiograms differed in susceptibility by more than three classes of antimicrobial agents. The PFGE patterns of computer and clinical isolates in the same ward (MRSA isolates from ward 8B and *A. baumannii *isolates from ward 11CI and 18 EN) were different that had similarity less than 70% (Fig. 1 and Fig. 2). However, interestingly, two *A. baumannii *computer isolates from ward 7C and one *A. baumannii *computer isolate from ward 15ESI2 had the same pulsotype (Figure 1).

## Discussion

This hospital-based surveillance study indicated the rate of MRSA and *A. baumannii *contamination of ward computers was 1.1% and 4.3%, respectively. No computer device was contaminated by *P. aeruginosa*. The MRSA contamination rate in our study was lower than that reported in a UK referral centre (1%) [18], but very different from that reported for a UK acute district general hospital (24%) [19]. This difference might be related to differences in hospital size, extent of computer use, and hand hygiene compliance. We supposed the relatively good hand hygiene compliance among our HCWs contributed to the lower contamination rate, although the two previous studies did not provide hand hygiene compliance data. The significant difference in the level of contamination of ward computers at different hospitals implies that computers can have very different roles as reservoirs of nosocomial pathogens. Compared with a high rate (37%) of environmental surfaces in patients' rooms harbor MRSA sampled from studies during outbreak and non-outbreak situations [20], ward computer interface surfaces seem to play a minor role as pathogen reservoirs.

Studies in ICUs indicate a more important role of computers there as pathogen reservoirs than do the computers of Non-ICU wards. Two previous studies have shown that contamination rate of computers in ICU workstations was as prevalent as that of the computers in patient rooms [8,21]. Computer interface surfaces in an ICU station were contaminated with potentially pathogenic microorganisms at a higher rate (6.3%) than the other surfaces [21]. However, for computer interfaces at ward stations, our results did not reveal a higher contamination rate in ICUs than that in non-ICUs.

Although contamination of inanimate surfaces by microorganisms has long been recognized, its impact on patients' infections is not clear [22]. The fact that forty-two percent of personnel had MRSA contamination on their gloves without entering the rooms of patients with MRSA infections suggests that contaminated environmental surfaces outside infected patients' rooms may increase the risk of MRSA transmission [3]. The association of clinical isolates and environmental contamination isolates was demonstrated for a 9-bed ICU, in which the researchers used PFGE typing of sequential isolates from clinical and environmental specimens [23]. An investigation of computer interface devices in an ICU indicated indistinguishable strains of MRSA from computers of patients' rooms, doctors' station, and clinical specimens [8]. Our hospital-based surveillance of MRSA by PFGE typing did not identify an association of MRSA isolates from computer interface devices and from patients. It is noteworthy that our study was different for a significantly lower rate of MRSA contamination (1.1%) than this previous study (11.1%) [8]. However, the identity of indistinguishable strains of *A. baumannii *from computer devices in two different wards (Figure 1) suggested the computer as a potential route of clonal spread in a hospital, although no subsequent clinical isolate was of this strain in our study.

Compared with the relatively low MRSA contamination rate (1.1%) on ward computer interface surfaces in our study, the 53% MRSA hand contamination rate among HCWs after contacting environmental surfaces near hospitalized patients in Bhalla A. et al study, showed that HCW's role as pathogen reservoirs and suggested hand hygiene to be important [2]. Beside to improve hand-hygiene compliance, improvement of cleaning services could be administered as an effective infection control measure [20]. Disinfectants including chlorine, alcohol, phenol and quaternary ammonium are all effective against MRSA, *P. aeruginosa*, and vancomycin-resistant *Enterococcus *species on keyboards of computers; and even sterile water is effective to remove more than 95% bacteria [5]. Flat keyboard with an alarm was suggested for being easy to clean and associated with better cleaning compliance [24]. Although keyboards can be safely and successfully disinfected and the need to clean computer interface surfaces as a routine practice is generally accepted, no specific cleaning and disinfection frequency and procedure for computer accessories has been defined [6,25]. Domestic cleaning has been reported useful to control MRSA [26]. As daily cleaning and hygiene regulation for using computer were demonstrated to be useful interventions to reduce keyboard contamination [9], several recommendations were gradually adopted, including that computers should be disinfected daily and when visibly soiled and HCW should not touch computer keyboards with contaminated hands [5]. Our result of the trend for the association of contamination rate of any of *S. aureus*, *Pseudomonas *spp. and *Acinetobacter *spp on the keyboards and mouse devices suggested both interface surfaces required attention when conducting cleaning service.

In contrast to previous studies on the role of environmental colonization that were performed during nosocomial pathogen outbreaks [27], our study was conducted when there was no outbreak. We did not investigate other factors in the transmission route, such as HCWs' hand carriage and colonization of patients. As sporadic MRSA strains differed in the shorter survival duration on environmental surface than outbreak strains [28], our study of a non-outbreak setting might be associated with the lower chance to find the similar genotype strains between computer and clinical isolates. Our study result is also limited by the cross sectional design that no measurement of the contamination levels was performed at two time periods with different hand hygiene compliance. Therefore it is difficult to make a conclusion about hand hygiene could help to prevent contamination of hospital ward computers, though inanimate environment surfaces in hospitals played a role among many steps for the transmission of pathogen to patients and hand hygiene promotion may be beneficial to reduce the risk of cross contamination [29].

## Conclusion

Our report documents that the contamination rates of computer interface surfaces by MRSA, *P. aeruginosa*, and *A. baumannii *in a hospital-wide surveillance were low when a relatively good hand hygiene compliance of HCWs was observed. Furthermore, no clinical correlation of contamination of these computer devices to clinical isolates was found. Routine disinfection and even surveillance of these computer devices may not be mandatory in non-outbreak settings.

## Competing interests

The authors declare that they have no competing interests.

## Authors' contributions

PLL and TPC designed the study. YHC and SFL advised on the clinical aspects of the study. LM and WGC participated in the laboratory work. PLL, LKS, YHC and CTC prepared the manuscript with major contributions from other authors. All authors read and approved the final version of the manuscript.

## Pre-publication history

The pre-publication history for this paper can be accessed here:

http://www.biomedcentral.com/1471-2334/9/164/prepub
